# Comparative transcriptomic analysis of wheat cultivars differing in their resistance to *Fusarium* head blight infection during grain-filling stages reveals unique defense mechanisms at play

**DOI:** 10.1186/s12870-023-04451-0

**Published:** 2023-09-16

**Authors:** Can Chen, Qi Guo, Qifang He, Zhuangbo Tian, Weihao Hao, Xinyu Shan, Jie Lu, Bronwyn J. Barkla, Chuanxi Ma, Hongqi Si

**Affiliations:** 1 Key Laboratory of Wheat Biology and Genetic Improvement On Southern Yellow and Huai River Valley, Ministry of Agriculture and Rural Affairs, College of Agronomy, Anhui Agricultural University, Hefei, 230036 China; 2 Faculty of Science and Engineering, Southern Cross University, Lismore, 2480 NSW Australia

**Keywords:** Pathogen defense, Immune response, Toxin accumulation, Cell wall, Biotic stress, Wheat head blight, Stress resistance

## Abstract

*Fusarium* head blight (FHB) is a devastating fungal disease that poses a significant threat to wheat production, causing substantial yield losses. Understanding the molecular mechanisms of wheat resistance to FHB is crucial for developing effective disease management strategies. This study aimed to investigate the mechanisms of FHB resistance and the patterns of toxin accumulation in three wheat cultivars, Annong8455, Annong1589, and Sumai3, with different levels of resistance, ranging from low to high respectively, under natural field conditions. Samples were taken at three different grain-filling stages (5, 10, and 15 DPA) for gene expression analysis and phenotypic observation. Results found that toxin concentration was inversely correlated with varietal resistance but not correlated with disease phenotypes, indicating that toxin analysis is a more accurate measure of disease status in wheat ears and grains. Transcriptomic data showed that Sumai3 exhibited a stronger immune response during all stages of grain filling by upregulating genes involved in the active destruction of pathogens and removal of toxins. In contrast, Annong1589 showed a passive prevention of the spread of toxins into cells by the upregulation of genes involved in tyramine biosynthesis at the early stage (5 DPA), which may be involved in cell wall strengthening. Our study demonstrates the complexity of FHB resistance in wheat, with cultivars exhibiting unique and overlapping defense mechanisms, and highlights the importance of considering the temporal and spatial dynamics of gene expression in breeding programs for developing more resistant wheat cultivars.

**Supplementary Information:**

The online version contains supplementary material available at 10.1186/s12870-023-04451-0.

## Introduction

Wheat (*Triticum aestivum*) is a staple food and industrial raw material that is a major dietary component of human and animal nutrition. Pathogens such as fungi, viruses, and bacteria can cause significant yield losses in wheat, averaging 21.5% globally [1]. *Fusarium* head blight (FHB), is a fungal disease that causes sterility and low thousand-grain weight, and ultimately yield losses of up to 80% in wheat [2, 3]. The fungus responsible for FHB, *Fusarium graminearum* infects the entire spike through the florets of wheat and causes single and multiple mycotoxin contamination, including deoxynivalenol (DON) and nivalenol (NIV) [4]. With changes in climate and cultivation practices, FHB has been expanding its range across China, shifting northward and westward from its traditional location in the middle and lower reaches of the Yangtze River, making Huang-Huai River wheat growing region susceptible [5]. Factors such as weather conditions, cultivation systems, grain harvesting methods all influence FHB infection. While some field management techniques to reduce infection, such as crop rotation with non-host crops, have been implemented, the control effect is limited [6]. Therefore, a deeper understanding of the FHB resistance mechanism in wheat is needed to provide more effective solutions.

Efforts have been made to reveal the genetic basis of FHB-resistant wheat cultivars, and over 400 quantitative trait loci (QTLs) have been identified [7]. However, genetic resistance against FHB is complex, quantitatively controlled, and significantly affected by environmental factors [8]. So far, only seven QTLs (*Fhb1*-*Fhb7*) are officially named and designated as behaving as Mendelized genes [9]. Among them, only *Fhb1* and *Fhb7* have been cloned, but the molecular mechanism of these FHB resistance QTLs still remains unclear.

Several genes have been identified that directly confer resistance to deoxynivalenol (DON) accumulation or DON detoxification in wheat, including a uridine diphosphate-glycosyltransferase (UGT) gene that conferred DON resistance in wheat by conjugating DON into less toxic DON-3-glucoside [10]; the trichothecene biosynthetic genes (the *TRI* genes) that were responsible for the 3-O-acetylation reaction that reduced in vitro activity significantly [11, 12]; the ribosomal protein L3 (*RPL3*) which confers resistance to mycotoxins by modifying the target site of mycotoxin DON [13, 14]; and virus-induced gene silencing of ATP-binding cassette (ABC) transporters identified in wheat, found to contribute to DON tolerance [15, 16].

Transcriptome analysis is a valuable tool in investigating the molecular mechanisms behind cereal resistance to fungal infections as the costs of this technique decrease and its applications become more widespread. For instance, RNA profiling of barley spikes showed that multiple defense mechanisms were activated in response to DON treatment as demonstrated by significant upregulation of gene transcripts encoding ABC transporters, UGT, cytochrome P450s, and glutathione-S-transferases [17]. In wheat, microarray analysis of spikelets from wheat near-isogenic lines revealed that the increased accumulation in gene transcripts encoding expansins and proline-rich proteins in the resistant cultivar resulted in cell wall modification, suggesting cell wall fortification may play a role in protecting the resistant cultivar [18]. A comparison of a highly resistant cultivar and a highly susceptible mutant inoculated with *F. graminearum* for 24 h and 48 h also showed that jasmonic acid pathways were associated with wheat resistance to pathogen infestation [19]. The expression of many of the induced genes varied between resistant and susceptible cultivars, indicating a wide range of basal defense responses [20].

During the flowering stage of wheat production, spikelet flowering occurs first in the middle of the head, followed by the ends. As the spikelets continue to flower, FHB infection gradually expands. During this time, toxins are produced and accumulate, a process that continues throughout the grain-filling period [21]. Some highly resistant wheat varieties exhibit low incidence of infestation, indicating that their resistance to invasion and expansion persists during the flowering and post-flowering stages. However, studies have shown that different inoculation positions on wheat spikes significantly influenced disease severity and mycotoxin accumulation in grains [22]. In addition, most transcriptomics studies have focused on early time points after FHB inoculation, typically within 96 h, which may not fully capture the toxin accumulation process of *F. graminearum*. Even less is known about the variability of toxin accumulation on wheat metabolism as FHB infestation progresses over time [23]. Furthermore, most of these studies used point-inoculation in controlled cabinet environments, resulting in consistent anther extrusion and infection times of each spikelet, masking the different mechanisms of disease resistance in different wheat cultivars, such as active destruction of pathogens and passive prevention of the spread of toxins into cells, highlighting the need for further research into FHB resistance in wheat under natural conditions.

In this study, three wheat cultivars with varying resistance levels to FHB were analyzed. Annong1589 is a new high-yielding cultivar with moderate resistance to diseases, including FHB, yellow rust, leaf rust, powdery mildew and sharp eyespot. Due to its low accumulation of DON and NIV, it is widely grown in Huang-Huai wheat growing region [24]. Sumai3 is one of the well-characterized sources of resistance to FHB, it is frequently used in breeding programs [25]. Annong8455, on the other hand, is highly susceptible to FHB [26]. This study focused on the accumulation of toxins within the grains during grain filling rather than the immediate host response to fungal invasion. The objective was to understand the process of toxin accumulation, which requires the presence of grains and a certain amount of time for the grain-filling process. Therefore, natural infection conditions allowed wheat spikes to be continuously infected by *Fusarium*, which means the plant constantly responds to this invasion. To capture different stages of plant development and assess how gene expression levels change over time during FHB infection, spikes from the three cultivars differing in resistance were collected at 5, 10, and 15 days post-anthesis (DPA) under natural infection conditions. By comparing the changes to the transcriptomes and measuring toxin accumulation of the three cultivars, this research aims to identify key pathways and genes associated with FHB resistance. The findings of this study will provide valuable insights into the molecular mechanisms underlying FHB resistance in wheat, which could ultimately aid in the development of new cultivars with enhanced resistance to this devastating disease.

## Results

### Spike characteristics of Sumai3, Annong1589 and Annong8455 after flowering under fungal Stress

**Fig. 1:**
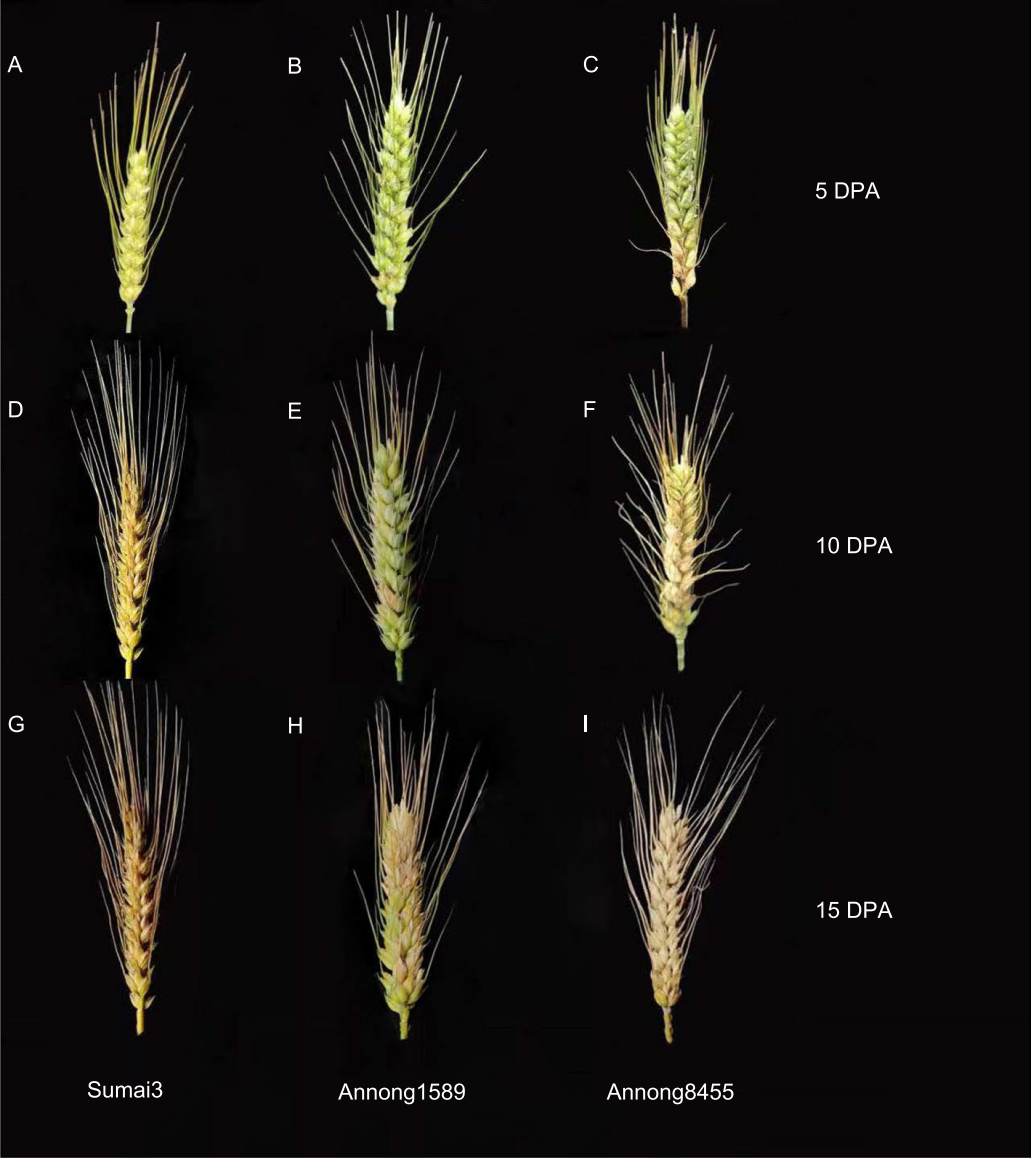
Spikes of three wheat cultivars with different resistance to FHB. **A** Sumai3(R), **B** Annong1589(M), **C** Annong8455(S) at 5 DPA. **D** Sumai3(R), **E** Annong1589(M), **F** Annong8455(S) at 10 DPA. **G** Sumai3(R), **H** Annong1589(M), **I** Annong8455(S) at 15 DPA

The spikes and grains of three wheat cultivars were monitored for signs of FHB infection at various timepoints after anthesis (Fig. 1). No visible fungal infection was observed on the spikes of Resistant cultivar Sumai3(R) (Fig. 1A) and Moderately resistant cultivar Annong1589(M) (Fig. 1B) at 5 days post-anthesis (DPA). However, several spikelets of the Susceptible cultivar Annong8455(S) showed infection (Fig. 1C). By 10 DPA, the infection area on spikelets of Annong8455(S) had increased and was observed on over half of the spikes (Fig. 1F), while only a slight infection was detected in the middle of the spikes in Annong1589(M) (Fig. 1E). At the last timepoint (15 DPA), the entire surface of the spikes of Annong8455(S) had been infected (Fig. 1I). The spikelet infection of Annong1589(M) increased and gradually expanded from the middle to the top and bottom of the spike (Fig. 1H). Meanwhile, no infection was observed on the spike and kernel of Sumai3(R). A noticeable contrast was observed in the spike color between Sumai3(R) and the Annong1589(M) and Annong8455(S), with Sumai3(R) exhibiting a golden color associated with gradual maturity, while Annong1589(M) and Annong8455(S) displayed a chalky pink color indicative of disease susceptibility. (Fig. 1D, 1G). Nevertheless, only a few kernels of Annong8455(S) turned red at 15 DPA, which is a typical phenotype of FHB infection, although the spike phenotype is easy to observe, the influence of FHB on the kernels is difficult to distinguish with the naked eye (Figure S1). Over time, the grains of the three cultivars gradually filled. The grains of Annong1589 (M) were significantly fuller compared to Sumai3(R) (Figure S1), consistent with the previous reports that the yield of Annong1589 is higher than Sumai3 [24, 27].

**Fig. 2:**
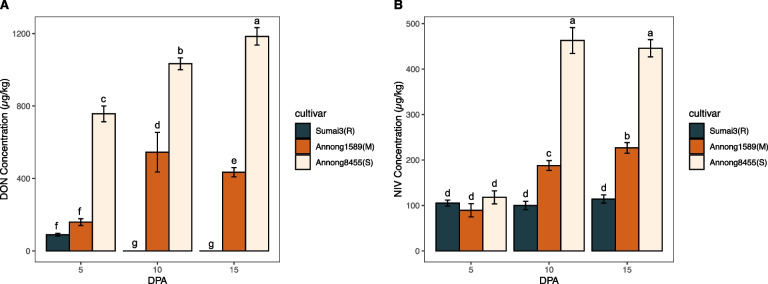
DON (**A**) and NIV (**B**) concentration in Sumai3(R), Annong1589(M) and Annong8455(S) at 5, 10 and 15 DPA. The means were compared using Duncan method [28]. Different letters in each figure represent the significant (*P*-value < 0.05) differences between cultivars and time points

Spikes of three wheat cultivars infected with FHB at 5, 10, and 15 DPA were collected and the levels of DON and NIV were measured. Annong8455(S) had the highest DON and NIV levels overall, followed by Annong1589(M). Sumai3(R) had the lowest levels of DON, while NIV at 5 DPA, was not significantly different from the other two cultivars (Fig. 2). In addition, the three cultivars exhibited different toxin accumulation patterns over time (Fig. 2). Sumai3(R) consistently had the lowest concentrations of both DON and NIV in the grains at 5, 10, and 15 DPA compared to the other cultivars, with DON only detected in the initial stages of infection. The DON accumulation of Annong1589(M) peaked at 10 DPA, which was four times higher than measured at 5 DPA. Levels then showed a decrease at 15 DPA (Fig. 2A). Annong8455(S) also showed an increase in NIV levels from 5 to 10 DPA. This pattern of toxin level changes was not consistent with either changes in spike or kernel phenotypes (Fig. 1 and S1).

### Overview of the transcriptomic datasets

Transcriptome profiling was performed on the three wheat cultivars: Sumai3(R), Annong1589(M) and Annong8455(S). Spikes were collected from each cultivar at three time points: 5, 10, and 15 DPA. RNA was obtained from these samples, and libraries were constructed for transcriptome sequencing by the Shanghai OE Biomedical Technology Co. (https://www.oebiotech.com/) Paired-end reads were pretreated using the next-generation sequencing quality control Toolkit (v2.3.3) software to remove sequences containing adapters or poly-N above 5%, and low-quality reads to produce valid data (clean data) for downstream sequence assembly [29]. Meanwhile, valid ratio, Q30 and GC contents were calculated for filtered clean data (Supplemental Table S1). The clean reads were compared to the reference wheat genome sequence published by the International Wheat Genome Sequencing Consortium (IWGSC). The total numbers of reads for each cultivar are listed in Supplemental Table S2, with total mapped reads ranging from 86.1% to 95.7%. Read counts were imported to the R studio for downstream analysis using the DESeq2 package. Principal component analysis (PCA) was performed using normalized counts of all samples.

### Expression patterns and identification of differentially expressed genes

**Fig. 3:**
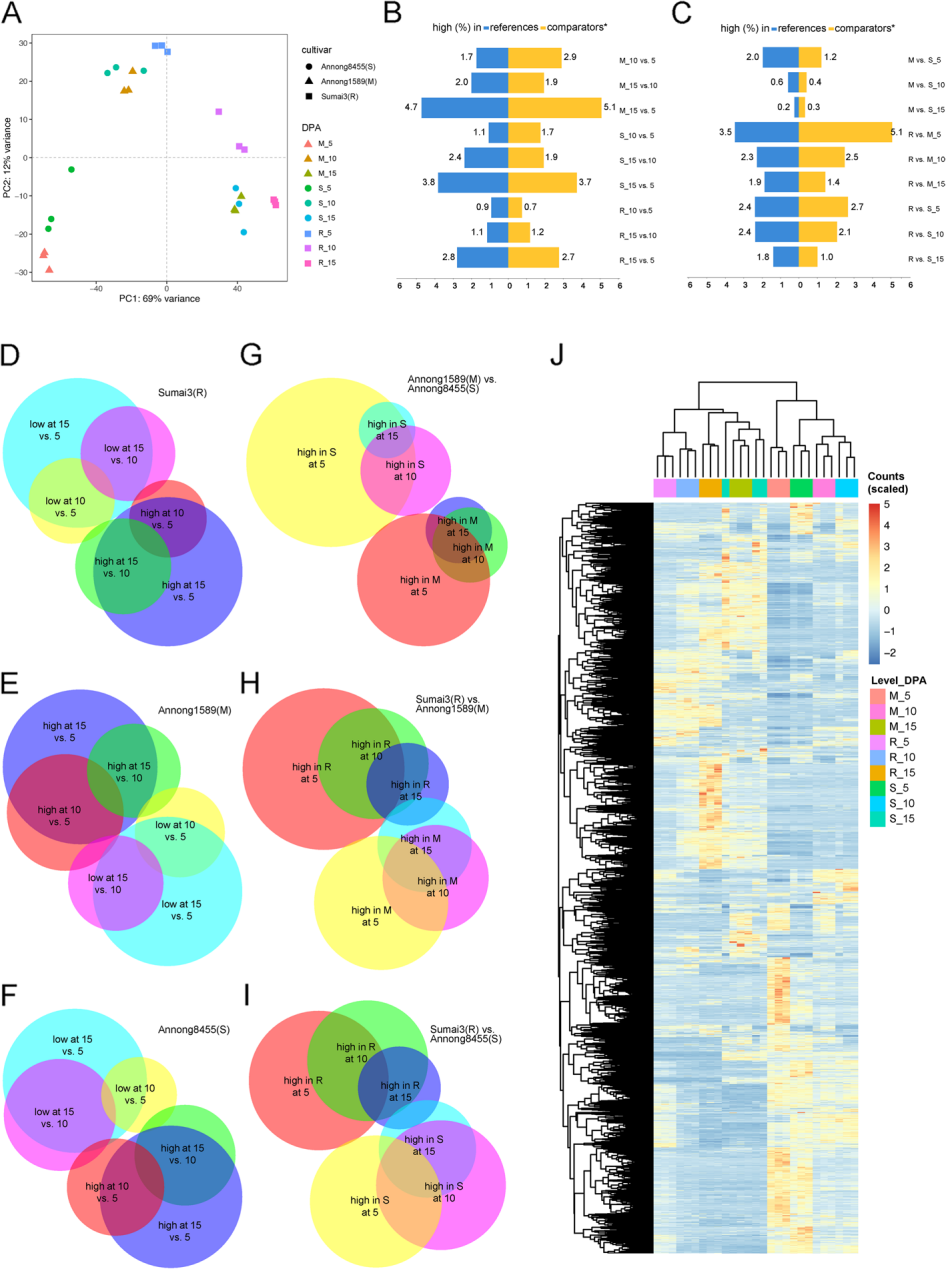
Transcriptomics of the spikes of three wheat cultivars collected at three different timepoints. **A** Principal component analysis (PCA) of the transcriptome of all biological samples (3 cultivars × 3 timepoints × 3 biological replicates) based on the normalized counts of all genes. Sample scores for the first and second principal components are plotted, with the explained percentage of variance of PC1 and PC2 indicated along the x and y axes. **B** and **C** Percentages of genes significantly differentially expressed in each comparison group. Genes were considered significantly changed if their log_2_fold change (FC) is greater than 2.5 or less than -2.5, and had an adjusted (Benjamini-Hochberg) *p*-value < 0.01. *In each comparison, the earliest time point or lowest FHB resistance was used as a reference. **D**-**I** Venn diagrams showing the gene overlaps between each comparison. **J** Hierarchical clustering heatmap comparing the differentially expressed genes in all studied samples

As shown in the Principle Component Analysis (PCA) in Fig. 3A, PC1 and PC2 together explained 81% of the variation in the transcriptome data. At 5 DPA, the cultivars showed complete segregation on the PCA plot, however, at 10 and 15 DPA, Annong1589(M) and Annong8455(S) clustered together but were separated from Sumai3(R). To identify differentially expressed genes (DEGs), pairwise comparisons were performed between the three cultivars for each time point. Genes with a log_2_ fold change (FC) greater than 2.5 or less than -2.5, and an adjusted *P*-value (Benjamini-Hochberg) < 0.01, were considered significantly up- or down-regulated (Supplemental Table S3).

The results indicated that the samples taken at 15 DPA had the most up- and down-regulated genes among all cultivars compared to those taken at 5 DPA (Fig. 3B). However, there were differences between cultivars. For example, the moderately resistant cultivar Annong1589(M) had the highest percentage of up- and down-regulated genes when comparing 5 to 15 DPA after flowering, accounting for 5.1% and 4.7% of the entire transcriptome, respectively. In contrast, Annong8455(S) and Sumai3(R) had lower percentages of DEGs over the experimental time period, with a total (up- and down-regulated together) of approximately 7.5% and 5.5% (Fig. 3B). The greatest differences were observed at five DPA for all comparisons when comparing different cultivars at the same sampling time. Among which, the disease-resistant cultivar Sumai3(R) and the moderately resistant cultivar Annong1589(M) had the highest percentage of DEGs, accounting for 8.6% of the total number of genes, including 5.1% upregulated and 3.5% down-regulated (Fig. 3C). The differences between Annong1589(M) and Annong8455(S), and between Sumai3(R) and Annong8455(S) at five days were lower, totaling 3.2% and 5.1%, respectively (Fig. 3C).

**Fig. 4:**
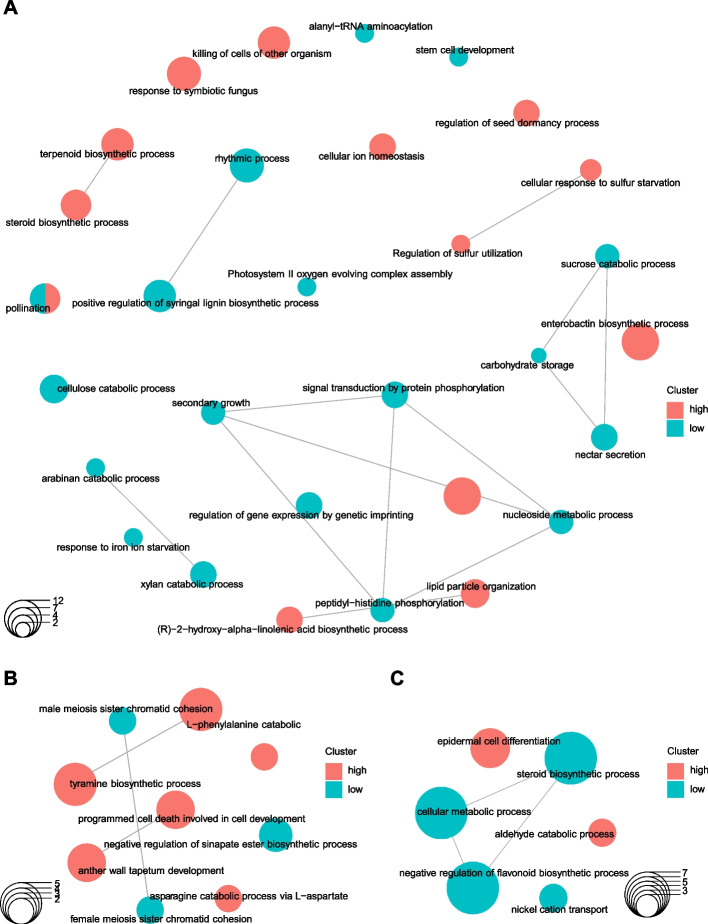
Gene Ontology (GO) enrichment maps of DEGs. Transcriptomic regulations in biological processes in **A**, Sumai3(R), **B**, Annong1589(M) and **C**, Annong8455(S) compared with the other two cultivars at 5 DPA after flowering. The size of the GO term circle reflects the number of genes enriched in the corresponding item. The lines connecting different nodes in the enrichment map indicate the overlapping gene sets and facilitate the identification of functional modules or clusters of gene sets that share common biological themes

Venn diagrams of DEGs revealed that for all three species, the genes that significantly changed at 15 DPA compared to 5 DPA can be considered as reflecting cumulative processes because most of the DEGs when comparing 5 to 10 DPA, and when comparing 10 to 15 DPA overlapped with those DEGs when 5 to 15 DPA were compared (Fig. 4D, E&F). A similar pattern was observed in the comparison between cultivars. The DEGs of 5 DPA played a dominant role in the two-by-two comparison of the three cultivars, because about half of the DEGs of 10 and 15 DPA overlapped with those at 5 DPA (Fig. 4G, H & I). A hierarchical clustering heatmap of the normalized counts of all DEGs (significantly regulated in at least one comparison) showed that there were two major clusters; in particular Sumai3(R) at all timepoints displayed the greatest differences compared to the other two cultivars (Fig. 4J). Although Annong1589(M) and Annong8455(S) were clustered together at 10 and 15 DPA, gene expression of Annong1589(M) at 5 DPA was distinct from Annong8455(S) (Fig. 4J).

### Pathway enrichment analyses

To further investigate the mechanisms that confer resistance to FHB in Sumai3(R) and partial resistance in Annong1589(M), Gene Ontology (GO) enrichment analyses were applied to DEGs (Supplemental Table S4). As samples collected at 5 DPA had the highest number of DEGs when comparing different cultivars (Fig. 3 G, H, & I), the GO terms that were enriched in those genes identified as significantly up- or down-regulated in each cultivar compared to the other two cultivars at 5 DPA were investigated. For instance, when compared with Annong1589(M) and Annong8455(S) at 5 DPA, Sumai3(R) displayed an active defense metabolism, as significant enrichment was observed in biological pathways relating to enterobactin biosynthetic process, killing of cells of other organisms, cellular response to sulfur starvation, cellular ion homeostasis, terpenoid biosynthetic process and steroid biosynthetic process (Fig. 4A). In addition, Sumai3(R) also likely had higher lipid anabolism, as genes involved in seed oilbody biogenesis and lipid particle organization were significantly enriched (Fig. 4A). However, the enrichment of genes involved in carbohydrate metabolism of Sumai3(R) at 5 DPA was significantly lower than those of the other two cultivars (Fig. 4A). For example, pathways such as carbohydrate storage, photosystem II oxygen evolving complex assembly, sucrose catabolic process, and stem cell development were significantly lower in Sumai3(R). In addition, Sumai3(R) showed a difference in cell wall metabolism, as positive regulation of syringal lignin biosynthetic process, cellulose catabolic process, arabinan catabolic process, and xylan catabolic process were enriched for significantly lower expressed genes (Fig. 4A). It is noteworthy that changes in pathways including killing of cells of other organisms, terpenoid biosynthetic process, regulation of sulfur utilization and photosystem II oxygen evolving complex assembly were consistently higher in Sumai3(R) compared to the other two cultivars throughout the 15-day period after flowering (Supplemental Table S4).

Intriguingly, as a moderately resistant cultivar, Annong1589(M) appears to employ a different mechanism of disease resistance compared to Sumai3(R), as highlighted by gene expression comparison at 5 DPA. Annong1589(M) was found to show enrichment in pathways related to tyramine biosynthetic process, and phenylalanine catabolic process, which are related to tyrosine decarboxylase activity and phenylacetaldehyde synthase activity (Fig. 4B). Organic compounds, such as phenylpropanoids synthesized by plants from the amino acids phenylalanine and tyrosine, have been shown to induce programmed cell death through the hypersensitivity reaction at the site of infection by incorporation of these compounds into cell walls of adjacent cells [30]. This is consistent with our results indicating the higher expression of genes involved in positive regulation of plant-type hypersensitive response and programmed cell death in Annong1589(M) at 5 DPA post infection (Fig. 4B). In contrast, the susceptible cultivar Annong8455(S) did not exhibit an elevated defense mechanism compared to Annong1589(M) and Sumai3(R). Rather, an enrichment in pathways related to aldehyde catabolic process, epidermal cell differentiation, lower cellular metabolic process and steroid biosynthetic process were observed in Annong8455(S) (Fig. 4C).

**Fig. 5:**
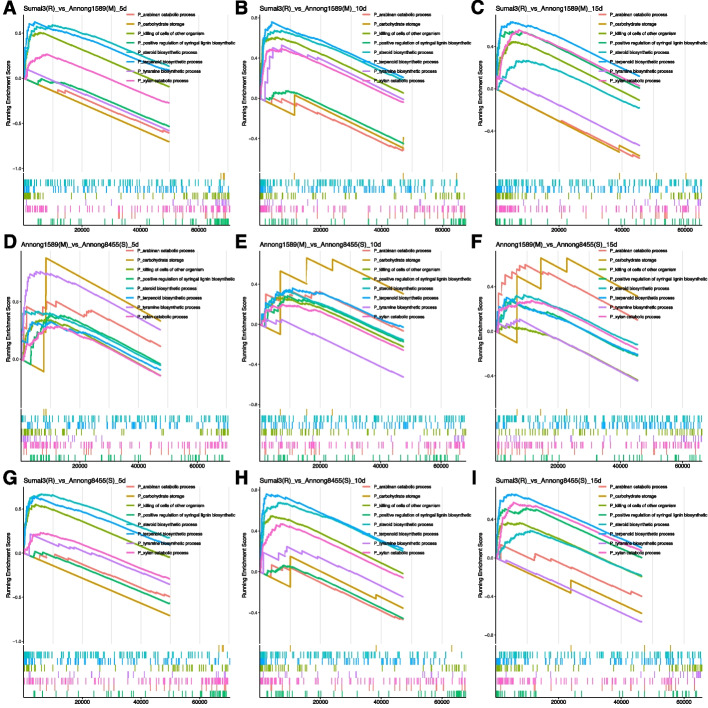
Gene set enrichment analysis (GSEA) and the expression comparison of the pathways of interest at transcriptional level. Sumai3(R) vs. Annong1589(M) at **A**: 5, **B**: 10 and **C**: 15 DPA. Annong1589(M) vs. Annong8455(S) at **D**: 5, **E**: 10 and F: 15 DPA. Sumai3(R) vs. Annong8455(S) at G: 5, H: 10 and I: 15 DPA. The FHB less resistant cultivar in the comparison was used as a reference. The x-axis of the enrichment plot represents the rank of the differential expression metric of genes in the dataset. The enrichment score (ES) line shows the trend of the enrichment score as the ranked gene list progresses. Positive values indicate enrichment at the top of the list, which indicate the genes in the gene set are more highly ranked and overrepresented, while negative values indicate enrichment at the bottom and inversely associated with the comparator

In order to study the transcriptome-wide differences among the three cultivars during the grain-filling stages, Gene Set Enrichment Analysis (GSEA) was also used to identify the overrepresented pathways in each comparison (Supplemental Table S5). Pathways including killing of cells of other organisms, terpenoid biosynthetic process, regulation of sulfur utilization, photosystem II oxygen evolving complex assembly, tyramine biosynthetic process and L-phenylalanine catabolic process are all enriched as shown in Fig. 5 and support the GO enrichment data (Fig. 4). Notably, Sumai3(R) had the highest expression of genes involved in terpenoid biosynthesis and steroid biosynthetic process at all three time points compared to the other two cultivars, while Annong8455(S) had the lowest expression, as indicated by the enrichment scores (ES) (Fig. 5).

The differences in the tyramine biosynthetic process were more pronounced in Annong1589(M) during grain grain-filling than in the other two cultivars. It is interesting to note that the relatively high expression of genes in this pathway in Annong1589(M) was high at 5 DPA (Fig. 5A & D), but showed no difference between the cultivars at 10 DPA (Fig. 5 B & E). Although the enrichment of this pathway in Annong1589(M) was again higher than that of Sumai3(R) at 15 DPA, it remained low compared to Annong8455(S) (Fig. 5 C& F). Sumai3(R) consistently exhibited significantly higher expression of genes involved in the killing of cells of other organisms pathway compared with both Annong1589(M) and Annong8455(S) (Fig. 5). Based on the results, it appears that the expression level of genes involved in carbohydrate storage was highest in Annong1589(M), followed by Annong8455(S), and lowest in Sumai3(R), and this pattern was maintained throughout the grain filling stage (Fig. 5 A-I).

### Validation of DEGs through RT-qPCR

**Fig. 6:**
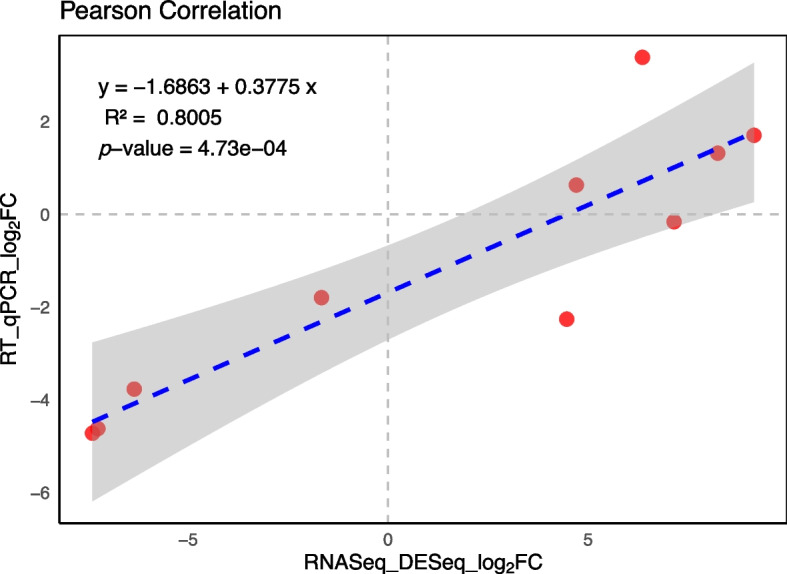
Comparison of the log_2_ fold change of selected transcripts using RNA-seq and RT-qPCR

To validate the RNA-seq results, a set of ten genes was selected for RT-qPCR. These genes were chosen based on their involvement in primary and secondary metabolism, active defense against pathogens, toxin removal, and passive prevention of toxin spread by strengthening the cell wall. The regulation of these genes, as measured by both RT-qPCR and RNA-seq, is listed in Table S6. The expression patterns of these genes, determined by RT-qPCR, exhibited a high correlation (R^2^ = 0.8005, *p*-value = 4.73e-04) with the results obtained from the RNA-seq analysis (Fig. 6). This strong correlation validates the precision and reliability of the RNA-seq analysis conducted in this study for identifying changes in the transcriptome.

## Discussion

*Fusarium* head blight (FHB), is a damaging plant disease that results in significant economic losses due to reduced yields and degraded grain quality. In addition, FHB can contaminate grains with mycotoxins, which pose serious health risks for humans and animals when ingested [31]. To combat FHB, it is crucial to develop or identify wheat cultivars that are resistant to the disease, with breeding programs benefitting from utilizing different cultivars with varying levels of resistance [32]. However, the genetics underlying FHB resistance are complex, and the mechanisms are not yet fully understood [33]. In this study, we investigated the differences in phenotype, toxin concentration, and gene expression at 5, 10 and 15 DPA in three wheat cultivars with varying levels of FHB resistance, which were naturally infected in a field environment. Our aim was to gain a deeper understanding of the complex interactions between host plants and the pathogen under natural conditions, which can ultimately lead to more effective breeding strategies and disease management practices. During the flowering stage of wheat production, the infection and subsequent accumulation of *Fusarium* toxins occurs continuously along the wheat spikes. The resistance to FHB of the cultivars Sumai3 (R), Annong1589(M), and Annong8455(S) varies, and was evident from the degree of infection in their respective spikes, and the extent of the disease progression by 15 DPA (Fig. 1 G-I). While Sumai3(R) and Annong1589(M) did not show signs of infection at 5 DPA, the amount of DON concentration varied (Figs. 1 and 2). Previous studies indicated that the relationship between accumulation of mycotoxins and the percentage of grains infected is not expected to follow a clear trend [8] as slightly infected kernels are not always detectable to the naked eye. This underscores the importance of measuring toxin concentration over scoring spike phenotype in evaluating FHB resistance. Furthermore, QTL studies have identified genes whose expression corresponded to lower DON located to chromosomes 3BL and 3DL of wheat, with minor or no effects on FHB disease index [34]. This suggests that toxin accumulation and spike phenotype may be controlled by different genes and involve different molecular mechanisms.

The transcriptome analysis showed that the differences in gene expression between the three wheat cultivars were most prominent at 5 DPA, as depicted in Fig. 3. Previous studies have suggested molecular mechanisms that may underlie the observed gene expression differences. For instance, the *Fhb1* gene is thought to encode a UGT or a regulator thereof, leading to the production of the less toxic DON-3-O-glucoside and reduced disease severity caused by FHB in wheat [35]. This finding offers a promising strategy for reducing DON accumulation by expressing appropriate UGT genes in transgenic plants. Additionally, some studies have reported the upregulation of cytochrome P450 genes in wheat and barley in response to DON application, with evidence suggesting that these genes may play a role in detoxifying DON [36]. Although direct evidence is currently lacking, a bacterial cytochrome P450 has been shown to catabolize DON to a less toxic derivative in wheat [37]. Furthermore, it has been suggested that proteins with antioxidant functions, such as glutathione S-transferases (GSTs), are upregulated in wheat at 5 d after inoculation following inoculation with *F. graminearum*. This upregulation may serve to reduce excessive levels of H_2_O_2_, thereby preventing cellular damage to lipids, proteins, and nucleic acids [16, 38]. In this experiment comparing three different resistant cultivars of FHB, it was found that these genes were indeed more highly expressed in the resistant cultivars (Supplemental Table S3), highlighting Sumai3(R)’s potential in reducing toxin accumulation (Fig. 2). But this difference is gradually diminished over time as shown by the data of the samples harvested at 10 and 15 DPA, which shows that the number of differentially expressed genes was reduced (Supplemental Table S3).

The GO enrichment analysis also revealed that Sumai3(R) exhibits a stronger active defense mechanism compared to the other two wheat cultivars. Of note, enterobactin is a siderophore that has a high affinity for iron and is commonly used by microbial systems to acquire this nutrient [39]. The observed high expression in enterobactin biosynthesis in Sumai3(R) may indicate a microbial nutrient acquisition pathway that mimics that of plants, potentially forming a nutritional competition between plants and fungi (Fig. 2). This process of nutrient competition via iron-chelating siderophores has been suggested to be antagonistic to *F. graminearum*, as it deprives the pathogen of a key resource [40].

Proteins involved in killing cells of other organisms (Fig. 4), including Knottin scorpion toxin-like domain-containing proteins (TraesCS1A02G014100, TraesCS1B02G018000, TraesCS1D02G012100), defensins (PDF21, TraesCS4D02G321100; PDF20 TraesCS5A02G496500), and chitin (TraesCS7A02G548000, TraesCS7B02G471500, TraesCS2B02G622200), are significantly higher in Sumai3(R) than in Annong1589(M) and Annong8455(S) at 5 DPA. This implies its enhanced ability to counteract FHB and reduce toxin levels (Fig. 2). Studies show that the scorpion toxin-like domain is mainly found in a subgroup of metazoan knottins from Arthropoda, which includes the antibacterial defensins [41]. Plant defensins and defensin-like proteins are antimicrobial peptides and are mostly involved in host defense. Antimicrobial peptides inhibit the growth of the phytopathogen *F. graminearum*, causing membrane permeabilization and cytoplasmic disorganization [42]. Furthermore, chitin in fungal cell walls can be hydrolyzed by chitinases into smaller oligomers or monomers, and play a significant role in plant-fungus pathogenic interactions [43]. Chitinase was found to be upregulated in FHB-resistant wheat Ning7840, and transgenic plants that overexpress chitinases exhibited enhanced resistance to pathogens [44]. Wheat that overexpressed a barley class II chitinase gene significantly increased Type II FHB resistance [45].

The significantly high gene enrichment in cellular ion homeostasis, terpenoid biosynthetic process, and steroid biosynthetic process in Sumai3 cells indicates their ability to maintain an intact cell structure and normal physiological metabolism (Fig. 4). In fact, recombinant inbred lines (RILs) of wheat carrying resistant alleles of *Fhb2* were found to have a higher abundance of metabolites belonging to the terpenoid biosynthetic pathway compared to susceptible RILs [46]. Some of these terpenoids have been identified to inhibit the growth of *F. graminearum* and trichothecene biosynthesis in barley [47]. Plant terpenoids are known to play a crucial role in defending against biological enemies either directly by acting as toxins or repellents for herbivores or indirectly by attracting predators or parasitoid enemies of herbivores [48]. However, it is difficult to identify the specific roles of individual terpenes due to the vast number and diversity of terpenes produced by plants. The diversity of specialized metabolites in plants is still not fully understood and remains a key goal in the study of plant defense mechanisms. While there are no studies on the mechanisms of sterols in crop resistance to FHB, it is known that exogenous application of sterols such as brassinosteroids can induce resistance against a broad range of diseases caused by fungal, bacterial, and viral pathogens by initiating innate immunity responses and maintaining cell membrane integrity [49, 50].

The significantly lower expression of genes involved in carbohydrate storage, photosystem II oxygen evolving complex assembly, and sucrose catabolic process in Sumai3(R) is consistent with its low-yielding species status, as illustrated in Supplemental Figure S1, where it can be seen that Sumai3(R) produces deflated and small seeds. On the contrary, we observed a significant enrichment of carbohydrate storage-related genes that were highly expressed in Annong1589(M), which exhibited fuller spikes and larger seeds (Fig. 1 and Supplemental Figure S1). These results agree with Annong1589(M) being considered a high yielding cultivar [24, 27]. The enrichment of arabinan and xylan catabolic process for significantly lower expressed genes, as shown in Fig. 4, may also contribute to the resistance of Sumai3(R) against pathogens. Specifically, arabinan chains play a key role in determining guard cell wall flexibility by maintaining fluidity within the pectin network in the walls, and *F. graminearum* arabinanase were found to enhance FHB susceptibility by suppressing wheat plant immunity [51]. Additionally, xylans are critical for maintaining the integrity of the plant cell wall and increasing cell wall recalcitrance to enzymatic digestion, which helps plants defend against pathogens. A study that produced transgenic wheat overexpressing a xylanase inhibitor demonstrated its ability to delay FHB blight disease symptoms significantly, indicating the importance of maintaining gene expression for these catabolic processes in Sumai3(R) for maintaining cell wall strength [52].

The GO term positive regulation of syringal lignin biosynthetic process was found to be significantly enriched in both Annong1589(M) and Annong8455(S) compared to Sumai3(R), which was surprising. Previous studies have shown transient silencing of NAC-like transcription factor genes in a resistant NIL wheat line reduced total lignin content and compromised FHB resistance. Increased lignin content is believed to help restrict the influx of *Fusarium* toxins and the efflux of plant nutrients [53]. This study suggested that the reinforcement of the cell wall in response to pathogens is divided into two types: active destruction of pathogens and passive prevention of infection. In Annong1589(M) genes involved in cell wall thickening were also upregulated, although this cultivar was not as resistant as Sumai3(R), which appears to be better at active defense.

Annong1589(M) also showed reduced disease susceptibility of wheat ears and seeds to a large extent compared to Annong8455(S) (Figs. 1 and 2), which could be related to the finding that genes related to tyramine biological process were enriched at 5 DPA in this cultivar. Studies have demonstrated that labeled tyramine accumulated in the cell walls of xylem cells in tobacco leaves, particularly in regions with peroxidase activity [54]. Previous research has also reported that cell wall thickening due to deposition of hydroxycinnamic acid amides (HCAAs) and tyramine-containing HCAAs was highly induced following pathogen inoculation in wheat [55]. Higher expression of tyrosine decarboxylases in Annong1589(M) is thought to contribute to not only pathogen defense by forming a physical barrier in cell walls, restricting the movement of the pathogen [56], but may also prevent cell wall degradation by the pathogen [57]. Additionally, the differential expression analysis revealed that Annong1589(M) had higher expression of genes involved in programmed cell death (Fig. 4), which can positively regulate plant resistance against pathogens [58]. During infection, biotrophic pathogens secrete effectors to interact with cognate resistant genes to induce programmed cell death and restrict pathogen spread in resistant hosts, which is a common mechanism in the gene-for-gene model of plant-pathogen interactions [59].

While the majority of pathways related to wheat resistance to FHB showed consistent differences among the three cultivars throughout the studied time periods,

variations were observed between the cultivars at different time points. For instance, significantly higher expression of genes involved in xylan catabolic process was observed in Annong1589(M) and Annong8455(S) at 5 DPA compared to Sumai3(R) at the same time point. However, at 10 and 15 DPA, Sumai3 had the highest expression of genes involved in this pathway (Figs. 4 and 5). It was previously suggested that the reduced xylan catabolic process in Sumai3(R) at 5 DPA may be linked to the maintenance of cell wall integrity, but studies have also shown that exogenous treatment of wheat spikes with xylanase leads to a reduction in *F. graminearum* infection symptoms and a lower fungal biomass accumulation, possibly due to the callose deposition detected in infected spikes, which is a common marker of plant defense response [60]. Therefore, we hypothesize that the delay of this defense mechanism to 10 DPA in Sumai3(R) may benefit from the expression of other immune mechanisms, such as the high expression of defensin and chitin genes under the category of killing of cells of other organisms (Fig. 4).

The moderately resistant cultivar, Annong1589(M), exhibited significantly higher levels of tyramine biosynthesis during this period, which was also observed in Sumai3(R) at 10 DPA, albeit to a lesser extent. The exact mechanism underlying tyramine's involvement in cell wall defense is not fully understood. Nevertheless, in addition to HCAAs, certain tyramine-based compounds, such as feruloyltyramine and 4-coumaroyltyramine, have also been shown to enhance disease resistance through their extracellular peroxidative polymerization, which is thought to strengthen cell walls [61, 62]. We speculate that FHB invasion may trigger upregulation of signaling pathways that regulate the tyramine pathway, as observed in Annong1589(M) at 5 DPA. This response occurred later at 10 DPA in sumai3(R), suggesting that sumai3(R) may have a more delayed passive defense response compared to Annong1589. Further research is required to unravel the precise mechanism underlying tyramine's role in FHB resistance during the flowering stage. Based on the phenotypic data and the number of differentially expressed genes among the cultivars at the three time points, it is evident that the early grain filling stage (5 DPA) plays a more crucial role in disease resistance (Figs. 3, 4 and 5).

The research findings offer valuable insights into the complex interactions between wheat cultivars and FHB, particularly focusing on toxin accumulation and defense mechanisms. The emphasis on toxin accumulation highlights the need for monitoring and managing mycotoxin levels in grains, which is crucial for both human health and grain marketability. Additionally, the distinction between active and passive defense mechanisms suggests that a multi-faceted approach, incorporating both genetic and environmental strategies, could be more effective in managing FHB.

### Experimental procedures

#### Plant materials and field assessment of FHB resistance

Three cultivars with different resistance to *Fusarium* head blight (FHB) were used in this study. Annong1589(M), a medium-gluten wheat cultivar developed at Anhui Agriculture University, exhibits moderate resistance to FHB [24]; Sumai3(R) is known for its high resistance to FHB and has been used in breeding programs worldwide [63]; while Annong8455(S) is highly susceptible to the fungus. The selected cultivars have closely aligned flowering and developmental periods to minimize potential discrepancies arising from varying flowering times [26]. Field assessment of FHB resistance was performed in the disease nursery of Anhui Agriculture University, Guohe, Anhui province (31°48′N, 117°23′E) during 2020–2021. The field site is recognized by Chinese National Wheat Improvement Center as a natural infection assessment nursery, for its unique characteristics such as high humidity and the specific climate of the Huang-Huai region, which makes it an ideal location for studying FHB disease. The cultivars were planted in a randomized block design in a plot consisting of three rows with a length of two meters and 20 cm spacing. The field trial was conducted under natural conditions with standard irrigation and fertilization practices, but no disease prevention measures. The incidence of FHB was assessed at 5, 10 and 15 days [64].

### Mycotoxin assay

During April–May 2021, wheat samples from Annong1589(M), Sumai3(R), and Annong8455(S) with different resistance to FHB, were collected at the grain filling stage at 5, 10, and 15 DPA (three biological replicates per cultivar per time point). Threshed wheat grains for toxin determination were dried in the oven for 72 h at 60 °C and sealed into airtight bags at room temperature (24 °C) for storage.

Multifunctional Column-Ultra Performance Liquid Chromatography-Diode Array Detector (MFC-UPLC-DAD) (Agilent Technologies 1290 infinityII; Agilent, Santa Clara, California, America) was used for simultaneous determination of DON and NIV fungal toxins concentration in wheat grains. A total of 25 g powdered wheat grain was weighed and added to 100 mL acetonitrile water (84:16, v/v) for extraction. The separation was performed on a C18 column (2.1 mm × 100 mm, 1.8 μm; Romer Labs, Beijing, China) after filtration and dilution by a multifunctional column (MFC) (MultiSep # 226; Romer Labs, Beijing, China). After filtration and dilution, the separation was carried out on a C18 column (2.1 mm × 100 mm, 1.8 μm) with acetonitrile-0.1% acetic acid water as mobile phase gradient elution. The diode array detector (DAD) was used for detection, and the external standard method was used for quantification [65]. Agilent OpenLAB CDS v2.2.0 software was used to data acquisition.

Statistical analysis. Data statistics and preliminary processing was carried out in Excel 2016. GraphPad Prism v5.0 was used to visualize the data. Based on the measured values of toxins in three cultivars and three periods, IBM SPSS Statistics 26 software was used to perform one-way ANOVA on the concentration of each toxin, and the Duncan method [28] was used for multiple comparison.

### RNA extraction, de novo assembly and sequencing

Three time periods (5DPA, 10 DPA, and 15 DPA) were selected for sampling after flowering for the three wheat cultivars of Sumai3(R), Annong8455(S) and Annong1589(M) and samplings were repeated three times for each period. The spikes were quickly cut off and frozen in liquid nitrogen, and stored at -80 °C. RNA extraction and cDNA libraries were carried outby Shanghai OE Biomedical Technology Co., Ltd following the protocol as described in the study published by L Zhou, Y Zong, L Li, S Wu, M Duan, R Lu, C Liu and Z Chen [66]. Total RNA was extracted from wheat seeds using mirVana miRNA isolation kit (Ambion, Austin, TX, USA) according to manufacturer 's protocol. RNA integrity was evaluated using an RNA-6000 Nano Kit of the 2100 Bioanalyzer (Agilent Technologies, Palo Alto, CA, USA). The quality of the constructed cDNA libraries was checked using a DNA-1000 Kit of the Bioanalyzer 2100 system (Agilent). The cDNA libraries were sequenced on an Illumina Novaseq 6000 platform (Illumina), and 150-bp paired-end reads were generated. Clean nucleotide sequence data ranged from 6.51 to 7.13 Gb, and the Q30 values were all > 92.4%, suggesting the data were reliable and of sufficient purity for further analysis. To remove low quality reads and sequence containing poly-N reads, raw data were processed by Trimmomatic v0.36. The BLASTX algorithm was used to search the IWGSC database (https://wheat-urgi.versailles.inra.fr/Projects/IWGSC) for Unigene annotation with a cut off e-value < 1e − 5. The GO (http://www.geneontology.org/) annotation of unigenes was determined by BLAST software.

The raw counts of mapped reads were filtered to exclude genes with low expression levels, retaining only those with a sum of at least 30 counts across all 27 samples (3 cultivars, each with 3 replicates at 3 time points). The differential gene expression (DEG) analysis was performed using the DESeq2 R package [67], which included normalization. Comparisons were made between different cultivars in the same period and between samples of the same cultivar taken at three different periods. DEGs were identified by applying the criteria of an adjusted *p-value* (the Benjamini–Hochberg method) less than 0.01 and a log_2_ fold change (FC) greater than 2.5 or less than -2.5. Results of this analysis can be found in Supplementary Table S3.

### Validation of RNA-Seq analysis by RT-qPCR

To validate the transcriptome results, RT-qPCR was performed on ten selected genes (Table S6) using the ABI 7500 Real-Time PCR System (Applied Biosystems, USA). The selected genes included GH16 domain-containing protein (TraesCS3B02G099000), Lipoxygenase (TraesCS2D02G528400), Aldehyde oxygenase (TraesCS4D02G093600), Chitinase (TraesCS7A02G548000, TraesCS7B02G471500), Defensin (TraesCS5A02G496500), Knottin scorpion toxin-like domain-containing protein (TraesCS1D02G012100, TraesCS1A02G014100), Cinnamyl-alcohol dehydrogenase (TraesCS2D02G422400), and Tyrosine decarboxylase (TraesCS3A02G410800). The primers for RT-qPCR analysis were designed using Primer Premier 5.0 (Table S6). Each reaction mixture had a final volume of 20 μL, comprising 2 × SybrGreen qPCR Master Mix (Sangon Biotech, Shanghai, China) at 1 µL, 10 μM forward primer at 0.2 µL, 10 μM reverse primer at 0.2 µL, 2 µL of cDNA, and 7.2 µL of ddH_2_O. The amplification process commenced with an initial denaturation step at 95 °C for 3 min, followed by 45 cycles of 95 °C for 15 s and 60 °C for 30 s. Subsequently, the melting curves of the RT-qPCR amplifications were obtained by gradually increasing the temperature from 60 °C to 95 °C. To ensure accurate normalization of gene expression levels, the gene actin of wheat was as the internal reference gene for RT-qPCR.

### Supplementary Information

**Additional file 1: Figuer S1.** The kernels of three wheat cultivars with different resistance to FHB at three timepoint.

**Additional file 2: Table S1.** Aligning statistics of clean reads with assembled unigenes.

**Additional file 3: Table S2.** Transcriptome reads and reference genome comparison rate statistics results.

**Additional file 4: Table S3-1.** Statistical analyses identifying differentially expressed genes A1589_T2_vs_T1.

**Additional file 5: Table S4-9.** Results of gene ontology enrichment analyses of differentially expressed genes Su3 vs other two_T3.

**Additional file 6: Table S5-9.** Gene Set Enrichment Analysis (GSEA) to identify the overrepresented pathways in each comparison Su3_vs_A1589_T3.

**Additional file 7: Table S6.** Selected genes and the their primers for RT-qPCR analysis*.

## Data Availability

The raw transcriptomic data generated in this study have been deposited to NCBI (https://www.ncbi.nlm.nih.gov/) and are available under BioProject PRJNA994698.
