# Supplementary material for: Comparative transcriptomic analysis of wheat cultivars differing in their resistance to Fusarium head blight infection during grain-filling stages reveals unique defense mechanisms at play
Source: BMC Plant Biol. 2023 Sep 16;23:433. doi: 10.1186/s12870-023-04451-0 (PMC10504723; doi:10.1186/s12870-023-04451-0)
Supplement: Supplementary file 1 — Additional file 1: Figuer S1. The kernels of three wheat cultivars with different resistance to FHB at three timepoint. [file 12870_2023_4451_MOESM1_ESM.pdf]

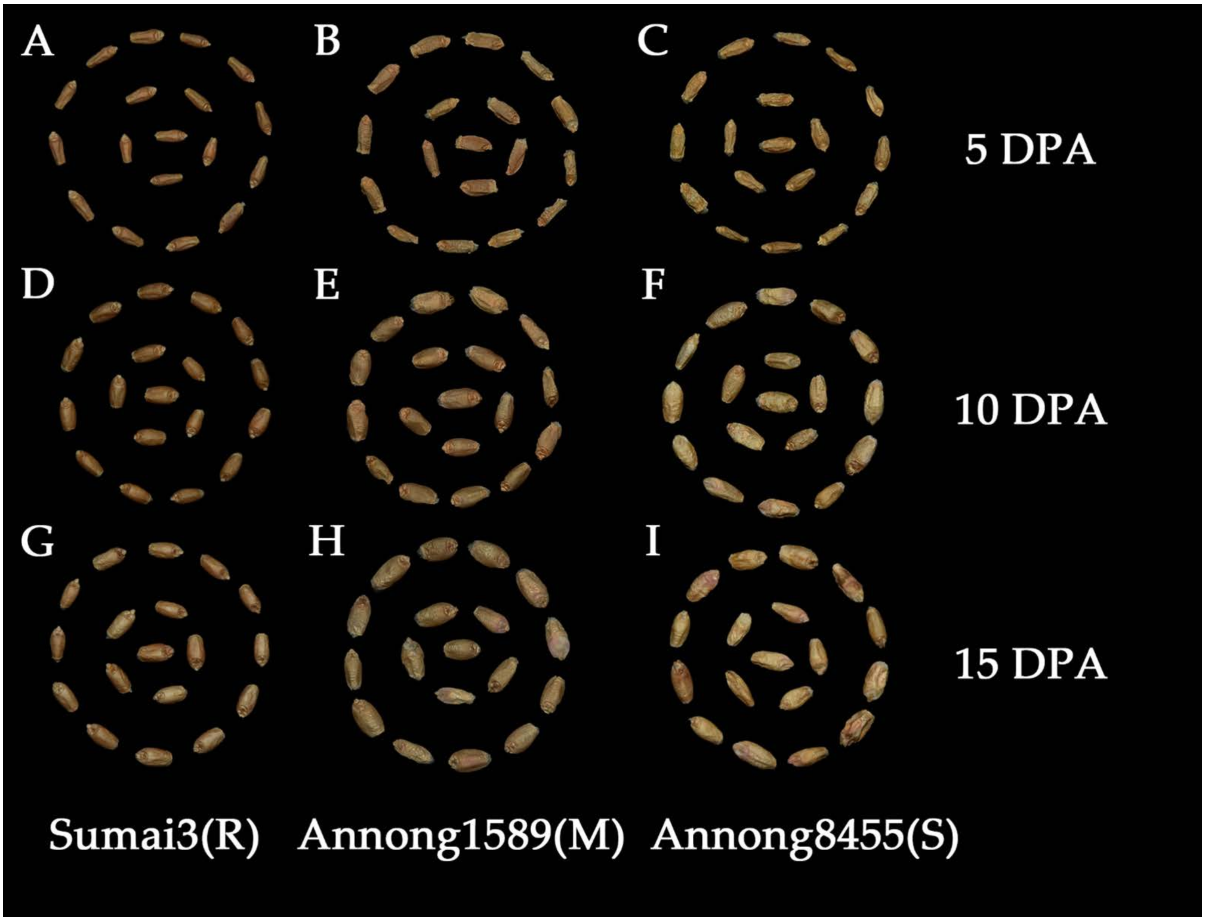

Figuer S1 The kernels of three wheat cultivars with different resistance to FHB at three timepoint.
